# Enteric-coated sodium bicarbonate supplementation improves high-intensity cycling performance in trained cyclists

**DOI:** 10.1007/s00421-020-04387-5

**Published:** 2020-05-09

**Authors:** Nathan Philip Hilton, Nicholas Keith Leach, Melissa May Hilton, S. Andy Sparks, Lars Robert McNaughton

**Affiliations:** 1 Department of Sport and Physical Activity, Sports Nutrition and Performance Research Group, Edge Hill University, St Helens Road, Ormskirk, L39 4QP UK; 2 Therapies Department, Liverpool Heart and Chest Hospital NHS Foundation Trust, Liverpool, UK; 3 Department of Sport and Movement Studies, Faculty of Health Science, University of Johannesburg, Johannesburg, South Africa

**Keywords:** Alkalosis, Extracellular buffering, Gastrointestinal symptoms, High-intensity exercise

## Abstract

**Purpose:**

Enteric-coated sodium bicarbonate (NaHCO_3_) can attenuate gastrointestinal (GI) symptoms following acute bicarbonate loading, although the subsequent effects on exercise performance have not been investigated. The purpose of this study was to examine the effects of enteric-coated NaHCO_3_ supplementation on high-intensity exercise performance and GI symptoms.

**Methods:**

Eleven trained male cyclists completed three 4 km time trials after consuming; a placebo or 0.3 g∙kg^–1^ body mass NaHCO_3_ in enteric-coated or gelatin capsules. Exercise trials were timed with individual peak blood bicarbonate ion concentration ([HCO_3_^–^]). Blood acid–base balance was measured pre-ingestion, pre-exercise, and post-exercise, whereas GI symptoms were recorded pre-ingestion and immediately pre-exercise.

**Results:**

Pre-exercise blood [HCO3^−^] and potential hydrogen (pH) were greater for both NaHCO_3_ conditions (*P* < 0.0005) when compared to placebo. Performance time was faster with enteric-coated (− 8.5 ± 9.6 s, *P* = 0.044) and gelatin (− 9.6 ± 7.2 s, *P* = 0.004) NaHCO_3_ compared to placebo, with no significant difference between conditions (mean difference = 1.1 ± 5.3 s, *P* = 1.000). Physiological responses were similar between conditions, although blood lactate ion concentration was higher with gelatin NaHCO_3_ (2.4 ± 1.7 mmol∙L^–1^, *P* = 0.003) compared with placebo. Furthermore, fewer participants experienced GI symptoms with enteric-coated (*n* = 3) compared to gelatin (*n* = 7) NaHCO_3_.

**Discussion:**

Acute enteric-coated NaHCO_3_ consumption mitigates GI symptoms at the onset of exercise and improves subsequent 4 km cycling TT performance. Athletes who experience GI side-effects after acute bicarbonate loading may, therefore, benefit from enteric-coated NaHCO_3_ supplementation prior to exercise performance.

## Introduction

High-intensity exercise bouts are impaired by peripheral fatigue (Thomas et al. 2015), typically as a result of disturbances to intramuscular homeostasis (Jones et al. 2008). Significant decreases in muscle and blood potential hydrogen (pH) have been reported (Hollidge-Horvat et al. 2000) as a result of the glycolytic contribution during high-intensity exercise (Baker et al. 2010; Gastin 2001). While the mechanisms responsible for the decline in muscular force across the neuromuscular junction are equivocal (Fitts 2016; Westerblad 2016), reductions in muscle pH are associated with simultaneous declines in muscle excitability (Cairns and Lindinger 2008), contractility (Spriet et al. 1989), glycolytic enzyme activity (MacLaren 1989), and exercise performance (Raymer et al. 2004). Exercise training and nutritional strategies that offset these perturbations to acid–base balance have, therefore, received considerable attention.

Inducing metabolic alkalosis prior to exercise, which can be achieved by oral ingestion of sodium bicarbonate (NaHCO_3_), has been shown to improve various performance measures (e.g., power, speed, and performance time) during single bouts of high-intensity exercise (Matson and Tran 1993; Peart et al. 2012; Lancha Junior et al. 2015). Through increases in extracellular bicarbonate ion concentration ([HCO_3_^–^]), NaHCO_3_ supplementation can augment buffering capacity (Siegler et al. 2010) and strong ion handling (Raymer et al. 2004), both of which favour high-intensity exercise performance. Although 0.2–0.4 g∙kg^–1^ body mass NaHCO_3_ is generally regarded as ergogenic during high-intensity exercise (McNaughton et al. 2016), gastrointestinal (GI) symptoms can be a problematic side-effect, with some individuals reporting severe symptoms (e.g., vomiting and diarrhoea) at the onset of exercise (Burke and Pyne 2007; Kahle et al. 2013). While some studies have shown that NaHCO_3_ can improve exercise performance despite GI distress (Price and Simons 2010), there is evidence to suggest that symptoms may compromise the performance-enhancing effects of supplementation (Cameron et al. 2010; Saunders et al. 2014; Deb et al. 2018). Furthermore, there is evidence to suggest that athletes may be deterred from supplementing with NaHCO_3_ due to the risk of GI symptoms during training and/or competition (Heibel et al. 2018).

Novel ingestion strategies are being investigated to alleviate GI symptoms, such as the administration of NaHCO_3_ in gastro-resistant capsules (Hilton et al. 2019a). Through the application of an enteric coating, which resists dissolution at a low pH (e.g., stomach), acid-sensitive ingredients such as NaHCO_3_ can bypass the stomach (Barbosa et al. 2017). Consequently, this reduces the neutralisation of gastric acid and minimises adverse side-effects (e.g., GI symptoms associated with elevated carbon dioxide tension in the GI tract). Indeed, delayed-release NaHCO_3_ has been shown to reduce the incidence and severity of GI symptoms compared with an aqueous solution, whilst increasing blood [HCO_3_^–^] and pH to comparable levels. In a recent study, enteric-coated NaHCO_3_ was shown to attenuate GI symptoms beyond encapsulation in gelatin and delayed-release capsules, which may be more favourable for those who experience GI symptoms post-ingestion (Hilton et al. 2019b). Nevertheless, changes in blood [HCO_3_^–^] and pH were lower with enteric-coated NaHCO_3_, potentially due to the absorption of bicarbonate across the intestinal mucosa (Turnberg et al. 1970) and less time available for absorption. Given that the degree of alkalosis can modulate the effects of NaHCO_3_ ingestion on exercise performance (Carr et al. 2011a), enteric-coated formulations may not favour performance improvements compared with alternative ingestion strategies. While enteric-coated NaHCO_3_ can reduce GI symptoms post-ingestion, no study to date has investigated the effects of supplementation on exercise performance. Therefore, it is unknown whether ingesting NaHCO_3_ in enteric-coated capsules alters the overall ergogenicity of supplementation. Furthermore, knowledge of the performance-enhancing potential of enteric-coated NaHCO_3_ would help to elucidate the impact of GI symptoms and acid–base balance on exercise performance, as well as improve the practical recommendations for athletes. The aim of the present study, therefore, was to determine whether enteric-coated NaHCO_3_ improves high-intensity exercise performance using an acute loading protocol.

## Methods

### Participants

Eleven trained male cyclists (according to DePauw et al. 2013) were recruited for the study (mean ± SD: age, 32 ± 12 years; body mass, 81.5 ± 12.5 kg; height 1.8 ± 0.1 m; peak oxygen uptake [$\dot{\mathrm{V}}$ O_2peak_], 63.2 ± 4.9 mL∙kg^–1^∙min^–1^) based upon sample size estimation. Sample size was determined a priori and revealed that 11 participants were required to detect changes (~ 3 s; 1.3%) in performance time between conditions with high statistical power (*α* = 0.05; *β* = 0.20). The benchmark for change in performance was chosen as it reflects the difference in performance time between podium and non-podium positions for similar cycling events (Christensen et al. 2017). All participants undertook regular cycling (≥ 3 day week^–1^) for at least 5 h week^–1^ and were free of GI-related disorders. Exclusion criteria included those with hypertension, renal impairment, or following a salt-restricted diet, and no participants were ingesting any nutritional supplements or medications at the time of the study. Ethical approval was obtained by the institutional research ethics committee and all participants gave written informed consent to take part in the study.

### Experimental design

In a randomised, double-blind, and crossover design, participants attended the laboratory on six occasions, separated by at least 48 h and at the same time of day (0900 h). During the initial visit, participants completed a preliminary test to determine $\dot{\mathrm{V}}$ O_2peak_ before familiarisation with the 4 km cycling time trial (TT). During the further two visits, individual responses to NaHCO_3_ ingestion (gelatin and enteric-coated) were established to determine subsequent ingestion timings. Throughout the next three visits, participants performed a maximal 4 km cycling TT under three different experimental conditions that were administered in a counterbalanced order. Experimental trials involved the consumption of 0.3 g kg^–1^ body mass of NaHCO_3_ in either enteric-coated or gelatin capsules, or a placebo containing cornflour prior to the 4 km TT. Participants were instructed to abstain from alcohol and caffeine consumption for 12 h, and strenuous exercise 24 h before each laboratory visit. Water intake was encouraged in the 24 h preceding experimental testing and participants were asked to arrive at the laboratory well hydrated and after an overnight fast to minimise the confounding effects of food intake on gastric emptying rates (Davis et al., 1986). On arrival to the laboratory, pre-test instructions were confirmed verbally to limit confounding nutritional effects on exercise performance. Physiological (heart rate and blood lactate) and perceptual responses were recorded throughout the 4 km TT, whereas acid–base balance and GI symptoms were recorded immediately pre- and post-exercise.

### Preliminary testing

Participants undertook an incremental exercise test to volitional exhaustion on an electromagnetically braked cycle ergometer (Lode Excalibur Sport, Groningen, The Netherlands) which confirmed that $\dot{\mathrm{V}}$ O_2peak_ was > 55 mL kg^–1^ min^–1^. The protocol involved a 5 min warm-up at 70 W and a self-selected cadence (70–120 rev min^–1^), after which the workload increased by 1 W every 2 s (30 W min^–1^) until volitional exhaustion. Breath-by-breath gases were measured continuously throughout using a gas analyser (Oxycon Pro™, Jaeger, Germany), whereas heart rate (Polar_®_, Kempele, Finland) and whole-body ratings of perceived exertion (RPE) were recorded each minute (Borg 1973). The following criteria were used to confirm that $\dot{\mathrm{V}}$ O_2peak_ had been reached: (1) heart rate within 10 beats min^–1^ of age-predicted maximum; (2) respiratory exchange ratio > 1.10 arbitrary units (AU); (3) RPE > 18/20 AU (Midgley et al. 2007). After a period of recovery (30 min), participants performed a 4 km cycling TT to familiarise themselves with the exercise protocol.

Individual responses to the ingestion of enteric-coated and gelatin NaHCO_3_ were established to allow exercise to be scheduled with peak bicarbonate buffering capacity. This method accounts for the inter-individual variability in acid–base kinetics following NaHCO_3_ ingestion (Jones et al. 2016) and differences between ingestion forms (Hilton et al. 2019b). Semi-nude body mass was recorded (Bod Pod_®_, Cosmed, Rome, Italy) after bladder evacuation to determine the dose of NaHCO_3_. Participants then consumed 0.3 g kg^–1^ body mass of NaHCO_3_ which was administered in either size 0 opaque enteric-coated (Bicarbi™, Nephcentric©, Arizona, USA) or gelatin capsules (Bulk Powders™, Colchester, UK). Enteric-coated capsules were pre-filled by the manufacturer, whereas gelatin capsules were manually filled by the researcher using a capsule filling device (Capsule Connection LLC, Arizona, USA). Given that each capsule contained 0.65 g of NaHCO_3_, supplements were administered to the nearest whole capsule. All supplements were checked for accuracy (Ohaus_®_, Fisher Scientific™, Pennsylvania, USA) prior to administration and were ingested with an equal volume (6 mL kg^–1^ body mass) of water (Evian_®_, Danone, Paris, France) within 5 min of commencing ingestion. Fingertip capillary blood samples (95 μL) were drawn pre-ingestion and then every 20 min for 180 min post-ingestion, with 10 min sampling from 80 to 140 min. Fingertip capillary blood samples were collected in heparin-coated glass capillary tubes (Radiometer Medical Ltd, Copenhagen, Denmark) using an aseptic technique and analysed immediately (Radiometer ABL800 BASIC, Copenhagen, Denmark) for blood [HCO_3_^–^] and pH.

### Experimental trials

Upon arrival to the laboratory, participants sat resting for 20 min before a baseline (pre-ingestion) capillary blood sample was taken. Participants then ingested either 0.3 g kg^–1^ body mass of NaHCO_3_ administered in gelatin or enteric-coated capsules, or a placebo. Opaque gelatin capsules were also used in the placebo trials and an equal number of capsules (39 ± 13 capsules) were given to mask the experimental conditions. Pre-exercise acid–base balance was determined with a further blood sample, after the pre-determined time-to-reach peak blood [HCO_3_^–^] had passed. All blood samples were analysed immediately for [HCO_3_^–^] and pH, as well as sodium ([Na^+^]), potassium ([K^+^]), and chloride ion ([Cl^–^]) concentrations.

### Time trials

Participants selected a preferred handlebar and saddle position which was then replicated for all other experimental trials. After a 5 min self-selected warm-up and 3 min rest, participants performed a maximal 4 km cycling TT on an electromagnetically braked cycle ergometer (Velotron Pro_®_, RacerMate™, Seattle, USA) from a static start. Participants were instructed to complete the TT as fast as possible and were free to change gears throughout, although gear ratios were fixed. Visual feedback of cadence, gearing, and distance travelled was provided on-screen, although participants were blinded from power output, speed, and time elapsed. Strong verbal encouragement was given by the same individual at regular (0.5 km) intervals throughout and no water was provided during the TT. All TTs took place under standardised laboratory conditions (temperature 18–20 °C, humidity 45 ± 5%) and a fan was placed 5 m in front of the cycle ergometer to promote evaporative cooling. Participants undertook a 5 min cool-down at a self-selected workload immediately after completion of the TT.

### Physiological and perceptual measures

During each TT, blood lactate ion concentration ([La^–^]) was measured pre- and post-exercise, and every 1 km throughout using a portable lactate monitor (Lactate Pro 2, Arkray, Japan). At the same time points, lower limb ratings of perceived exertion (RPE-L) and RPE were recorded using a 6–20 scale (Borg 1973), whereas perceived ratings of fatigue (ROF) were recorded on a 10-point Likert scale (Micklewright et al. 2017). Heart rate was measured pre- and post-exercise, and every 1 km throughout the TT (Polar_®_, Kempele, Finland). Symptoms of GI distress were recorded immediately pre-exercise using an adapted GI symptom questionnaire (Carr et al. 2011b) including nausea, flatulence, stomach cramping, belching, stomach ache, bowel urgency, diarrhoea, vomiting, and stomach bloating. Symptoms were self-measured on a 10 cm visual analogue scale where “0 = No symptom” and “10 = Severe symptom” (Miller et al. 2016). Symptom terminology was explained to participants before the experimental trials commenced to ensure consistency in the reporting of symptoms.

### Statistical analyses

Data normality was assessed using the Shapiro–Wilk test and by visual inspection of the normality plots (Grafen and Hails 2002). One-way analysis of variance (ANOVA) for repeated measures were used to compare performance time and GI symptom scores. All performance (i.e., power), acid–base balance (i.e., blood [HCO_3_^–^] and pH), electrolyte ([Na^+^], [K^+^], and [Cl^–^]), physiological (i.e., blood [La^–^] and heart rate), and perceptual (i.e., RPE, RPE-L, and ROF) variables were analysed using two-way (condition × time) ANOVA for repeated measures. Where a significant main effect was revealed, Bonferroni-adjusted post hoc paired comparisons were determined (Atkinson 2002). Effect sizes were reported as eta-squared (*η*^2^) for one- and two-way ANOVA, whereas Hedge’s *g* and 95% confidence intervals (CI) were calculated for paired comparisons (Lakens 2013). Effects were discussed in relation to the relevant literature (Thompson 2007) and described as small (*η*^2^ = 0.01; *g* = 0.2), medium (*η*^2^ = 0.06; *g* = 0.5), or large (*η*^2^ = 0.14; *g* = 0.8) as previously suggested (Cohen 1988). Statistical significance was set at *P* < 0.05 and values for *P* of “0.000” given by the statistical package were corrected to “ < 0.0005” (Kinnear and Gray 1995). Descriptive data are presented as mean ± standard deviation (SD) throughout. Data were analysed using the Statistical Package for the Social Sciences version 25 software (IBM_®_, Chicago, USA), whereas sample size was calculated using GPower_®_ version 3.1.9.2 (Faul et al. 2007).

## Results

### Exercise performance

**Fig. 1:**
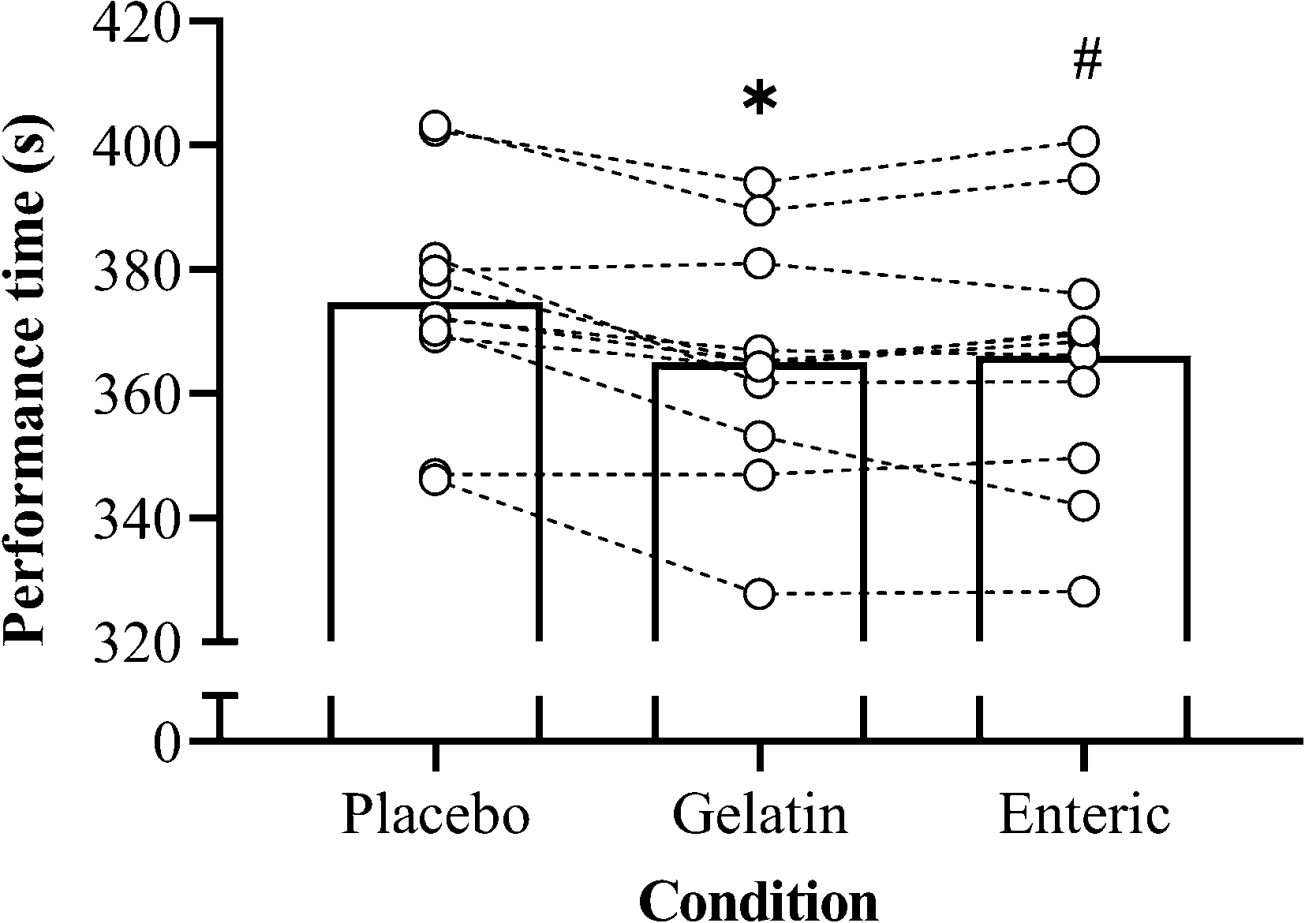
Mean ± SD 4 km TT performance time following the ingestion of 0.3 g·kg^–1^ body mass NaHCO_3_ in gelatin or enteric-coated capsules, or a placebo. Dotted lines denote individual performance times. *Significant difference between gelatin NaHCO_3_ and placebo (*P* < 0.05). ^#^Significant difference between enteric-coated NaHCO_3_ and placebo (*P* < 0.05)

There was a significant improvement in performance time (Fig. 1) in the NaHCO_3_ trials compared with the placebo (*F*_2.0, 20.0_ = 10.6, *P* = 0.001, *η*^2^ = 0.52). Performance time was significantly faster with enteric-coated (mean difference = 8.5 s [− 2.3%], *P* = 0.044, 95% CI [0.2, 16.9 s], *g* = 0.4) and gelatin (mean difference = 9.6 s [− 2.6%], *P* = 0.004, 95% CI [3.4, 15.9 s], *g* = 0.5) NaHCO_3_ compared with the placebo, but there was no difference between enteric-coated and gelatin NaHCO_3_ (mean difference =  − 1.1 s, *P* = 1.00, 95% CI [–5.7, 3.5 s], *g* = 0.1).

**Fig. 2:**
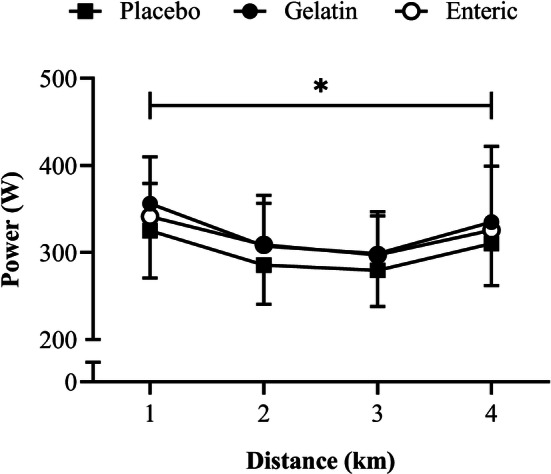
Mean ± SD power output following the ingestion of 0.3 g·kg^–1^ body mass NaHCO_3_ in gelatin or enteric-coated capsules, or a placebo. *Significant difference between gelatin NaHCO_3_ and placebo (*P* < 0.05)

Acute bicarbonate loading had a significant effect on power output (*F*_2.0, 20.0_ = 8.8, *P* = 0.002, *η*^2^ = 0.10; Fig. 2), with higher values during the gelatin trial when compared with the placebo (mean difference = 24 W [+ 7.7%], *P* = 0.023, 95% CI [3, 45 W], *g* = 0.5). No further differences in power output were shown between trials (*P* > 0.05). There was a significant variation in power output across the TT (*F*_1.4, 14.1_ = 12.8, *P* = 0.002, *η*^2^ = 0.34) with power output declining between 1 and 2 km (*P* = 0.001) before reaching a plateau (*P* = 0.123) at 3 km, followed by an increase towards 4 km (*P* = 0.026). Pacing strategies were similar between conditions (Fig. 2), with no significant condition × time interaction (*F*_2.6, 26.1_ = 0.4, *P* = 0.746, *η*^2^ = 0.01). No order effect on TT performance was shown given that neither performance time nor power output differed between the first and the last trials (all *P* > 0.05).

### Acid–base balance

**Fig. 3:**
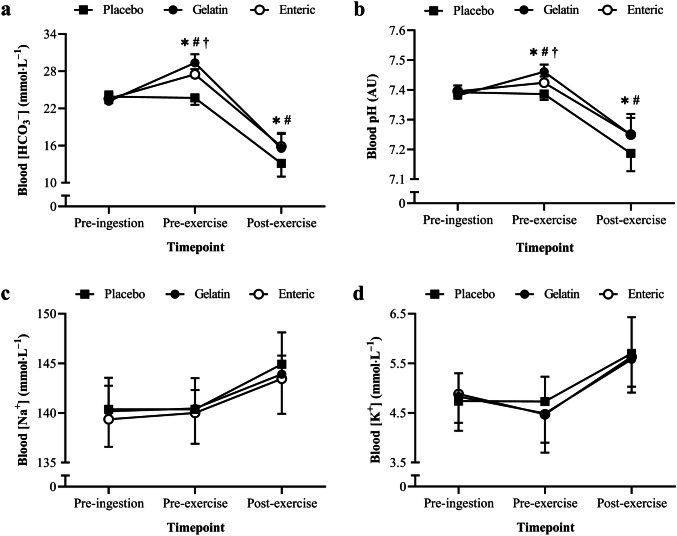
Mean ± SD blood **a** [HCO_3_^–^], **b** pH, **c** [Na^+^], and **d** [K^+^] pre-ingestion, pre-exercise (post-ingestion), and post-exercise. ^*^Significant difference between gelatin NaHCO_3_ and placebo (*P* < 0.05). ^#^Significant difference between enteric-coated NaHCO_3_ and placebo (*P* < 0.05). ^†^Significant difference between gelatin and enteric-coated NaHCO_3_ (*P* < 0.05)

The time-to-reach individual peak blood [HCO_3_^−^] was 110 ± 20 min (range 80–140 min) and 90 ± 20 min (range 60–130 min) in the enteric-coated and gelatin conditions, respectively. Blood [HCO_3_^–^] was significantly higher in the NaHCO_3_ conditions compared with the placebo (*F*_2.0, 20.0_ = 23.5, *P* < 0.0005, *η*^2^ = 0.04, Fig. 3a), with no difference between enteric-coated and gelatin capsules (*P* = 1.0). Blood [HCO_3_^–^] increased pre-exercise (*P* < 0.0005) followed by a decrease post-exercise (*P* < 0.0005), with a condition × time interaction (*F*_4.0, 40.0_ = 48.2, *P* < 0.0005, *η*^2^ = 0.87). Pre-exercise blood [HCO_3_^–^] was significantly higher in the enteric-coated (3.8 ± 1.0 mmol L^–1^, *P* < 0.0005, 95% CI [3.0, 4.7 mmol∙L^–1^], *g* = 3.8) and gelatin (5.6 ± 1.5 mmol∙L^–1^, *P* < 0.0005, 95% CI [4.3, 6.3 mmol∙L^–1^], *g* = 4.3) conditions compared with the placebo. Furthermore, blood [HCO_3_^–^] was significantly lower with enteric-coated compared with gelatin capsules pre-exercise (mean difference = 1.8 mmol∙L^–1^, *P* = 0.012, 95% CI [0.4, 3.3 mmol∙L^–1^], *g* = 1.5).

Blood pH was significantly higher in the NaHCO_3_ conditions compared with the placebo (*F*_2.0, 20.0_ = 14.6, *P* < 0.0005, *η*^2^ = 0.04, Fig. 3b), with no difference between enteric-coated and gelatin capsules (*P* = 1.0). Blood pH increased pre-exercise (*P* < 0.0005) followed by a decrease post-exercise (*P* < 0.0005), with a condition × time interaction (*F*_2.0, 19.7_ = 48.2, *P* = 0.001, *η*^2^ = 0.03). Pre-exercise blood pH was significantly higher in the enteric-coated (0.038 ± 0.016 AU, *P* < 0.0005, 95% CI [0.024, 0.052 AU]) and gelatin (0.074 ± 0.019 AU, *P* < 0.0005, 95% CI [0.058, 0.091 AU]) conditions compared with the placebo. Blood pH was also significantly lower with enteric-coated compared with gelatin capsules pre-exercise (mean difference = 0.037 AU, *P* = 0.001, 95% CI [0.018, 0.055 AU], *g* = 1.6).

### Electrolyte responses

Acute bicarbonate loading did not alter blood [Na^+^] (*F*_2.0, 20.0_ = 1.0, *P* = 0.394, *η*^2^ = 0.02, Fig. 3c), although there were significant increases shown post-exercise (*F*_2.0, 20.0_ = 20.5, *P* < 0.0005, *η*^2^ = 0.42). No condition × time interaction was shown for blood [Na^+^] (*F*_4.0, 40.0_ = 0.3, *P* = 0.850, *η*^2^ = 0.01). Similarly, NaHCO_3_ ingestion did not alter blood [K^+^] (*F*_2.0, 20.0_ = 0.2, *P* = 0.848, *η*^2^ = 0.01, Fig. 3d) despite significant increases post-exercise (*F*_2.0, 20.0_ = 41.1, *P* < 0.0005, *η*^2^ = 0.48), with no condition × time interaction (*F*_4.0, 40.0_ = 0.6, *P* = 0.660, *η*^2^ = 0.01).

### Physiological and perceptual responses

**Fig. 4:**
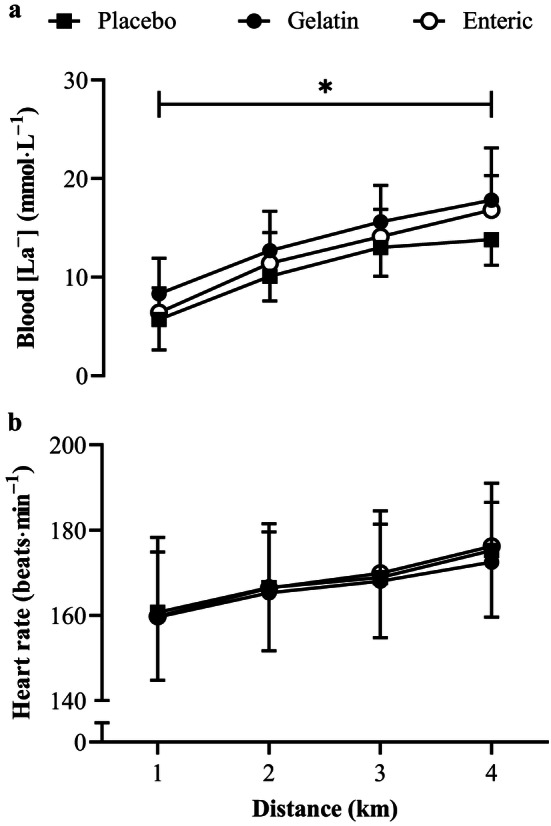
Mean ± SD **a** blood [La^–^] and **b** heart rate responses during the 4 km TT following the ingestion of 0.3 g·kg^–1^ body mass NaHCO_3_ in gelatin or enteric-coated capsules, or a placebo. ^*^Significant difference between gelatin NaHCO_3_ and placebo (*P* < 0.05)

Blood [La^–^] was significantly greater (*F*_2.0, 20_ = 7.7, *P* = 0.003, *η*^2^ = 0.03; Fig. 4a) in the gelatin trial compared with the placebo (mean difference = 2.4 mmol∙L^–1^, *P* = 0.003, 95% CI [0.9, 3.8 s], *g* = 0.9). No further differences in lactate responses were shown between conditions (*P* > 0.05), although blood [La^–^] progressively increased during all TTs (*F*_1.4, 13.9_ = 127.3, *P* < 0.0005, *η*^2^ = 0.82), without a condition × time interaction (*F*_2.5, 25.2_ = 2.0, *P* = 0.152, *η*^2^ = 0.01). Heart rate progressively increased throughout the 4 km TT (*F*_1.1, 10.9_ = 43.8, *P* < 0.0005, *η*^2^ = 0.60; Fig. 4b), although no significant differences were shown between conditions (*F*_2.0, 20_ = 0.7, *P* = 0.491, *η*^2^ = 0.01), nor was there a significant condition × time interaction (*F*_2.3, 22.5_ = 1.0, *P* = 0.385, *η*^2^ = 0.01). Despite improvements in TT performance in both NaHCO_3_ conditions, there were no differences in neither RPE (*F*_2.0, 20.0_ = 2.2, *P* = 0.137, *η*^2^ = 0.04), RPE-L (*F*_2.0, 20.0_ = 0.2, *P* = 0.841, *η*^2^ = 0.01) nor ROF (*F*_2.0, 20.0_ = 3.5, *P* = 0.05, *η*^2^ = 0.03) between conditions, although there were significant increases in RPE (*F*_3.0, 30.0_ = 63.2, *P* < 0.0005, *η*^2^ = 0.56), RPE-L (*F*_1.4, 14.4_ = 45.2, *P* < 0.0005, *η*^2^ = 0.53), and ROF (*F*_1.2, 12.4_ = 2.2, *P* < 0.0005, *η*^2^ = 0.67) during the TT (Table 1). No significant condition × time interactions were revealed for neither RPE (*F*_6.0, 60.0_ = 0.9, *P* = 0.524, *η*^2^ = 0.01), RPE-L (*F*_6.0, 60.0_ = 0.4, *P* = 0.893, *η*^2^ = 0.01) nor ROF (*F*_6.0, 60.0_ = 0.8, *P* = 0.583, *η*^2^ = 0.01).

**Table 1:** Mean ± SD perceptual responses during the 4 km TT

	Condition		
	Placebo	Gelatin	Enteric
RPE (AU)			
1-km	11.6 ± 1.9	11.5 ± 2.8	12.5 ± 2.0
2-km	13.3 ± 2.2	12.4 ± 2.5	14.0 ± 1.7^a^
3-km	14.8 ± 2.2^a^	13.8 ± 1.8	15.2 ± 2.1^a^
4-km	16.6 ± 2.3^a^	16.0 ± 2.8^a^	16.6 ± 2.2^a^
RPE-L (AU)			
1-km	13.5 ± 2.7	13.6 ± 2.2	13.7 ± 2.1
2-km	14.7 ± 2.5	14.6 ± 2.2^a^	15.0 ± 1.7
3-km	16.1 ± 1.9^a^	15.9 ± 2.0^a^	16.2 ± 1.6^a^
4-km	17.3 ± 2.4	17.8 ± 1.7^*^	18.0 ± 2.1^*^
ROF (AU)			
1-km	3.9 ± 1.8	3.2 ± 1.3	3.5 ± 1.1
2-km	5.0 ± 1.4^a^	4.7 ± 0.9^a^	5.2 ± 1.0^a^
3-km	5.8 ± 1.1^a^	5.4 ± 1.2	6.2 ± 1.3
4-km	7.5 ± 1.3^a^	6.6 ± 1.2^a^	7.5 ± 1.4^a^

*significant difference from the previous timepoint (*P* < 0.05)

### Gastrointestinal symptoms

No GI symptoms were reported pre-ingestion in all conditions. No participants reported GI symptoms pre-exercise with the placebo, whereas fewer participants experienced symptoms with enteric-coated (*n* = 3) compared to gelatin (*n* = 7) NaHCO_3_. Pre-exercise GI symptom scores were significantly higher following gelatin NaHCO_3_ (3.6 ± 3.9 AU) compared with placebo (*P* = 0.043), with no difference between enteric-coated NaHCO_3_ (1.0 ± 1.7 AU) and placebo (*P* = 0.324). Furthermore, pre-exercise GI symptoms were less severe with enteric-coated NaHCO_3_ compared to gelatin at the individual level (Table 2), although group symptom scores were similar (*P* = 0.211) between enteric-coated and gelatin capsules (mean difference = 2.6 AU, *P* = 0.211, 95% CI [1.1, 6.2 AU]).

**Table 2:** Individual GI symptom scores immediately before exercise. Symptoms are displayed in bold for clarity and scores are displayed in parentheses

	Condition		
Participant	Placebo	Gelatin	Enteric
1	No symptom (0.0)	No symptom (0.0)	No symptom (0.0)
2	No symptom (0.0)	**Diarrhoea (10.0)**	No symptom (0.0)
3	No symptom (0.0)	**Stomach ache (1.3)**	No symptom (0.0)
4	No symptom (0.0)	**Stomach cramp (1.5)**	No symptom (0.0)
5	No symptom (0.0)	No symptom (0.0)	**Flatulence (5.0)**
6	No symptom (0.0)	**Diarrhoea (6.0)**	No symptom (0.0)
7	No symptom (0.0)	**Bloating (5.0)**	**Bloating (3.0)**
8	No symptom (0.0)	No symptom (0.0)	No symptom (0.0)
9	No symptom (0.0)	**Bowel urgency (5.0)**	No symptom (0.0)
10	No symptom (0.0)	**Diarrhoea (10.0)**	**Bloating (2.0)**
11	No symptom (0.0)	No symptom (0.0)	No symptom (0.0)

## Discussion

This is the first study to investigate the effect of enteric-coated NaHCO_3_ supplementation on exercise performance, specifically that which would typically benefit from extracellular buffering agents. The main finding of this study was that ingesting enteric-coated NaHCO_3_ prior to exercise improved (~ 2.3%) subsequent 4 km cycling TT performance among trained cyclists. Despite inducing a lower degree of metabolic alkalosis with enteric-coated NaHCO_3_ (Fig. 3), there were no differences in exercise performance compared with a standard ingestion form (i.e., gelatin capsules). Furthermore, enteric-coated NaHCO_3_ reduced GI symptoms experienced immediately before exercise compared with gelatin capsules (Table 2), although subjective ratings of GI symptoms in this sample were low. When taken together, these data suggest that enteric-coated NaHCO_3_ improves high-intensity cycling performance in those with mild-to-moderate GI symptoms. However, the effects of enteric-coated NaHCO_3_ on exercise performance could be greater in those who experience more severe GI symptoms at the onset of exercise, although this warrants further investigation. Enteric-coated NaHCO_3_ supplementation may, therefore, offer an alternate strategy to improve high-intensity exercise performance and mitigate GI symptoms associated with acute bicarbonate loading.

Numerous studies have investigated the effects of NaHCO_3_ on simulated high-intensity TT events with equivocal outcomes (Callahan et al. 2017; Gough et al. 2018). Where some studies have reported performance improvements (Gough et al. 2018), others have reported no benefit (Callahan et al. 2017; Correia-Oliveira et al. 2017) following supplementation. This disparity between studies could be explained by the timing of supplementation, given that the current study demonstrated positive outcomes when exercise was timed with peak alkalosis. Studies that have reported no effect of NaHCO_3_ ingestion during similar exercise protocols have administered the supplement at a standardised time (Callahan et al. 2017; Correia-Oliveira et al. 2017) despite considerable variability in the time taken to reach metabolic alkalosis (Jones et al. 2016). Time between ingestion and the onset of exercise largely determines the degree of metabolic alkalosis in terms of blood [HCO_3_^–^] and pH (Heibel et al. 2018), which, in turn, may influence the ergogenicity of NaHCO_3_ supplementation (Carr et al. 2011a). Interestingly, the effect of NaHCO_3_ on exercise performance in the present study was mediated by the ingestion form, with a small-to-moderate effect on performance time (2.3–2.6%) with enteric-coated and gelatin NaHCO_3_, respectively. The present study reported a mean 5.6 mmol L^–1^ increase in blood [HCO_3_^–^] with gelatin compared to placebo, which is lower than the 3.8 mmol L^–1^ increase shown with the enteric-coated capsules. This finding is consistent with the previous studies that have investigated the acid–base kinetics following NaHCO_3_ ingestion (Hilton et al. 2019b), which could account for the difference in effect size reported in the present study. Nevertheless, exercise performance still improved with enteric-coated NaHCO_3_ supplementation, which questions the 5–6 mmol L^–1^ threshold suggested to improve performance (Carr et al. 2011a; Heibel et al. 2018). Furthermore, the improvements in 4 km cycling TT performance in the present study are similar to the previous studies, despite higher pre-exercise blood [HCO_3_^–^] reported by others (Gough et al. 2018). Given this disparity between studies, it is unlikely that timing is the only factor modulating the ergogenicity of NaHCO_3_ during high-intensity exercise.

Whilst an individualised ingestion strategy may increase the likelihood of commencing exercise with greater blood buffering capacity, it is not clear whether this optimises the ergogenicity of NaHCO_3_ supplementation. Individualising the timing of supplementation may also not be practical at present, for some athletes, given that this requires access to a blood-gas analyser. In the current study, however, mean ingestion timings corresponded to those that have been previously suggested with enteric-coated NaHCO_3_ (Hilton et al. 2019b). Furthermore, it is important to note that enteric-coated capsules delay the time-to-reach peak blood [HCO_3_^–^] following NaHCO_3_ ingestion, suggesting that the current recommendations (e.g., 60–90 min before exercise) are not appropriate for this ingestion form. Instead, the current study adds to the growing body of evidence, suggesting that enteric-coated NaHCO_3_ should be ingested ~ 120 min prior to exercise to maximise blood [HCO_3_^–^] if a standardised ingestion timing strategy is adopted (Hilton et al. 2019b). Whilst participants ingested the capsules in a fasted state in the present study, co-ingestion with food may delay gastric emptying and alter the release of NaHCO_3_ (Davis et al. 1986). Further research should look to compare the effects of an individualised and standardised ingestion time on subsequent performance, including the effects of prandial state on acid–base responses and GI symptoms following NaHCO_3_ ingestion.

Given that enteric-coated NaHCO_3_ improves exercise performance among those with mild-to-moderate GI symptoms, the effects on exercise performance may be enhanced among those with more severe GI symptoms at the onset of exercise. Indeed, GI distress was significantly reduced in some individuals in the current study (Table 2), although numerous individuals did not report symptoms at the onset of exercise. Although ergogenic doses (~ 0.3 g kg^–1^ body mass) of NaHCO_3_ may induce GI symptoms, these may not necessarily be timed with exercise performance. This is consistent with the previous studies (Hilton et al. 2019a, b) demonstrating the reduced incidence of GI symptoms at the time of peak alkalosis, despite severe symptoms at other timepoints. It is, therefore, difficult to elucidate whether GI symptoms can negate the ergogenic effects of NaHCO_3_ supplementation from the current data, since the overall incidence and severity of GI symptoms was low. Nevertheless, GI symptoms may hinder high-intensity exercise performance or dampen the ergogenic effects of NaHCO_3_ supplementation (Saunders et al. 2014). Further research should, therefore, examine the effects of enteric-coated NaHCO_3_ supplementation in those who typically report moderate-to-severe GI symptoms at the onset of exercise, as the effects may be greater among these individuals. Given that only few participants reported GI symptoms following enteric-coated NaHCO_3_ supplementation, future studies could consider increasing the dose (> 0.3 g·kg^–1^ body mass), which may also increase blood [HCO_3_^–^].

Whilst psychological indicators of perceived exertion and fatigue increased during exercise, no differences were reported between the placebo and NaHCO_3_ conditions (Table 1), suggesting an alternative mechanism other than reductions in afferent feedback to the central nervous system (Siegler and Marshall 2015). Nevertheless, this finding indicates the enhancements in power output were attained at a relatively similar RPE when supplementing with NaHCO_3_. Similarly, despite distinct changes in blood [Na^+^] and [K^+^] during exercise, no differences were shown between NaHCO_3_ and placebo (Fig. 3). Changes in these strong ions can impair muscle excitability (Cairns and Lindinger 2008), therefore, suggesting that improvements in performance were not due to ionic shifts in [Na^+^] and [K^+^] associated with enhanced contractility. Nevertheless, enhanced muscle contractile function cannot be dismissed as a potential mechanism, as altered calcium handling can improve mechanical efficiency (Siegler et al. 2016), although this cannot be elucidated from the current study. Alternatively, given that pre-exercise blood [HCO_3_^–^] and pH were greater in the NaHCO_3_ conditions compared to placebo, the performance improvements shown in the current study may be attributed to increases in extracellular buffering capacity. Reinforced extracellular concentrations of bicarbonate are suggested to promote H^+^ efflux from intramuscular to extracellular regions through increases in monocarboxylate transporter activity, which maintains muscle pH during exercise (Bishop et al. 2006). Given the delayed onset of intramuscular acidosis, NaHCO_3_ promotes glycolytic enzyme activity and flux, as indicated through increases in muscle glycogen utilisation and lactate concentrations (Hollidge-Horvat et al. 2000; Siegler et al. 2016). Although muscle pH and lactate were not measured in the current study, increases in muscle pH and lactate efflux have been shown during exercise following NaHCO_3_ supplementation (Costill et al. 1984). Augmenting glycolytic flux may have, therefore, permitted exercise at higher intensities and could explain the performance improvements reported in the current study. This would account for the greater blood [La^–^] shown with gelatin NaHCO_3_, although the increases reported with enteric-coated capsules did not reach significance (Fig. 4a). Given that monocarboxylate transporters 1- and 4 are stimulated by the intra- to extracellular [H^+^] gradient, the greater extracellular pH shown with gelatin capsules may have upregulated the co-transport of H^+^ and lactate to a greater extent, and could account for differences in the ergogenic effect size (0.3%). This may also explain why power output was greater when NaHCO_3_ was given in gelatin capsules (Fig. 2), although this did not result in greater overall performance times compared to enteric-coated capsules. Therefore, the current evidence suggests that while pre-exercise blood [HCO_3_^–^] does not determine the overall ergogenicity of NaHCO_3_ supplementation, the magnitude of such effects may be increased by a greater degree of metabolic alkalosis.

In summary, this study is the first to demonstrate that 0.3 g∙kg^–1^ body mass of enteric-coated NaHCO_3_ improves high-intensity exercise performance when timed with peak alkalosis. This study also provides novel data, highlighting that ingestion form (e.g., gelatin or enteric-coated capsules) can mediate the effects on exercise performance, potentially through the degree of induced alkalosis. To understand the implications of GI symptoms on exercise performance, further research should compare the effects of enteric-coated NaHCO_3_ supplementation on exercise performance in those who experience severe symptoms immediately before exercise, particularly as GI distress may be ergolytic among these individuals. Furthermore, given the growing range of ingestion forms commercially available to athletes (e.g., liquid, gelatin capsules, and enteric-coated capsules), future studies should compare the effects on exercise performance. Nonetheless, acute enteric-coated NaHCO_3_ consumption improves 4 km cycling TT performance and, therefore, may offer an appropriate ergogenic strategy for those who experience GI side-effects following supplementation.

## Abbreviations

[Cl^–^]: Chloride ion concentration
[H^+^]: Hydrogen ion concentration
[HCO_3_^–^]: Bicarbonate ion concentration
[K^+^]: Potassium ion concentration
[La^–^]: Lactate ion concentration
[Na^+^]: Sodium ion concentration
AU: Arbitrary units
CI: Confidence intervals
*g*: Hedge’s *g*
GI: Gastrointestinal
H^+^: Hydrogen ion
NaHCO_3_: Sodium bicarbonate
pH: Potential hydrogen
ROF: Rating of perceived fatigue
RPE: Rating of perceived exertion
RPE-L: Localised rating of perceived exertion
SD: Standard deviation
TT: Time trial
$\dot{\mathrm{V}}$O_2peak_: Peak oxygen uptake
*η*^2^: Eta-squared
